# Cuticular drusen associated with aneurysmal type 1 neovascularization (polypoidal choroidal vasculopathy)

**DOI:** 10.1186/s40942-018-0148-5

**Published:** 2018-12-05

**Authors:** Serena Fragiotta, Talia R. Kaden, K. Bailey Freund

**Affiliations:** 1 Vitreous Retina Macula Consultants of New York, 460 Park Ave., New York, NY 10022 USA; 2 LuEsther T. Mertz Retinal Research Center, Manhattan Eye, Ear and Throat Hospital, New York, NY USA; 3 Department of Ophthalmology, New York University School of Medicine, New York, NY USA; 4 Department of Medico-Surgical Science and Biotechnologies, Sapienza University of Rome, Rome, Italy; 5 Department of Ophthalmology, Manhattan Eye, Ear and Throat Hospital, New York, NY USA

**Keywords:** Cuticular drusen, Polypoidal choroidal vasculopathy, Aneurysmal type 1 neovascularization, Optical coherence tomography angiography, Fluorescein angiography, Indocyanine green angiography

## Abstract

**Background:**

Aneurysmal type 1 neovascularization (AT1) is a term recently introduced to better describe the aneurysmal dilatation that may arise from neovascular lesions, more commonly known as polypoidal choroidal vasculopathy. The proposed term, AT1, includes an expanded clinical spectrum of aneurysmal (polypoidal) lesions observed in both different ethnicities and associated with varied clinical phenotypes.

**Case presentation:**

A 61-year-old woman of European descent was referred for a new, asymptomatic retinal hemorrhage found on routine examination. Ophthalmoscopy revealed cuticular drusen in both eyes best appreciated on fundus autofluorescence, and a hemorrhagic retinal pigment epithelium detachment above the superior arcade in the right eye. In the fellow eye, a reddish appearing pigment epithelial detachment was noted nasal to the optic nerve. Indocyanine green angiography showed findings of AT1 in both eyes. Optical coherence tomography angiography showed intrinsic flow signal within the aneurysmal lesions.

**Conclusions:**

Eyes with cuticular drusen may develop AT1 which, to our knowledge, has not been described. This is an important observation because the documented coexistence of AT1 in the setting of a variant of age-related macular degeneration lends supports to this new understanding of AT1 as a growth pattern of neovascular tissue proliferating between the RPE and Bruch membrane, rather than as a distinct disease entity.

## Background

Cuticular drusen were first described in 1977 by Gass [1]. Like soft drusen, cuticular drusen are dynamic, with periods of absorption and coalescence, and they can be associated with neovascular age-related macular degeneration [2]. Recently, Dansingani et al. [3] proposed the term “aneurysmal type 1 neovascularization” (AT1) as a generic descriptor to describe neovascularization with aneurysmal features that is located between the RPE and the inner collagenous layer of Bruch’s membrane. They noted that following the original description of idiopathic polypoidal choroidal vasculopathy (PCV) as a polyp-like vascular structure originating from the inner choroid and occurring mostly in middle-aged black women, further experience has revealed that these aneurysmal lesions are in fact located within neovascular tissue proliferating above Bruch’s membrane. Additionally, as a variety of other retinal diseases occurring in all races may present with this neovascular pattern, the authors suggested the more descriptive term “aneurysmal type 1 neovascularization” to describe this entity [3–5]. Since eyes with cuticular drusen may develop macular neovascularization [2], it logically follows that AT1 might occur in this setting as well. To our knowledge this association has yet to be described. Herein, we report a patient presenting with bilateral AT1 in association with cuticular drusen.

## Case report

**Fig. 1:**
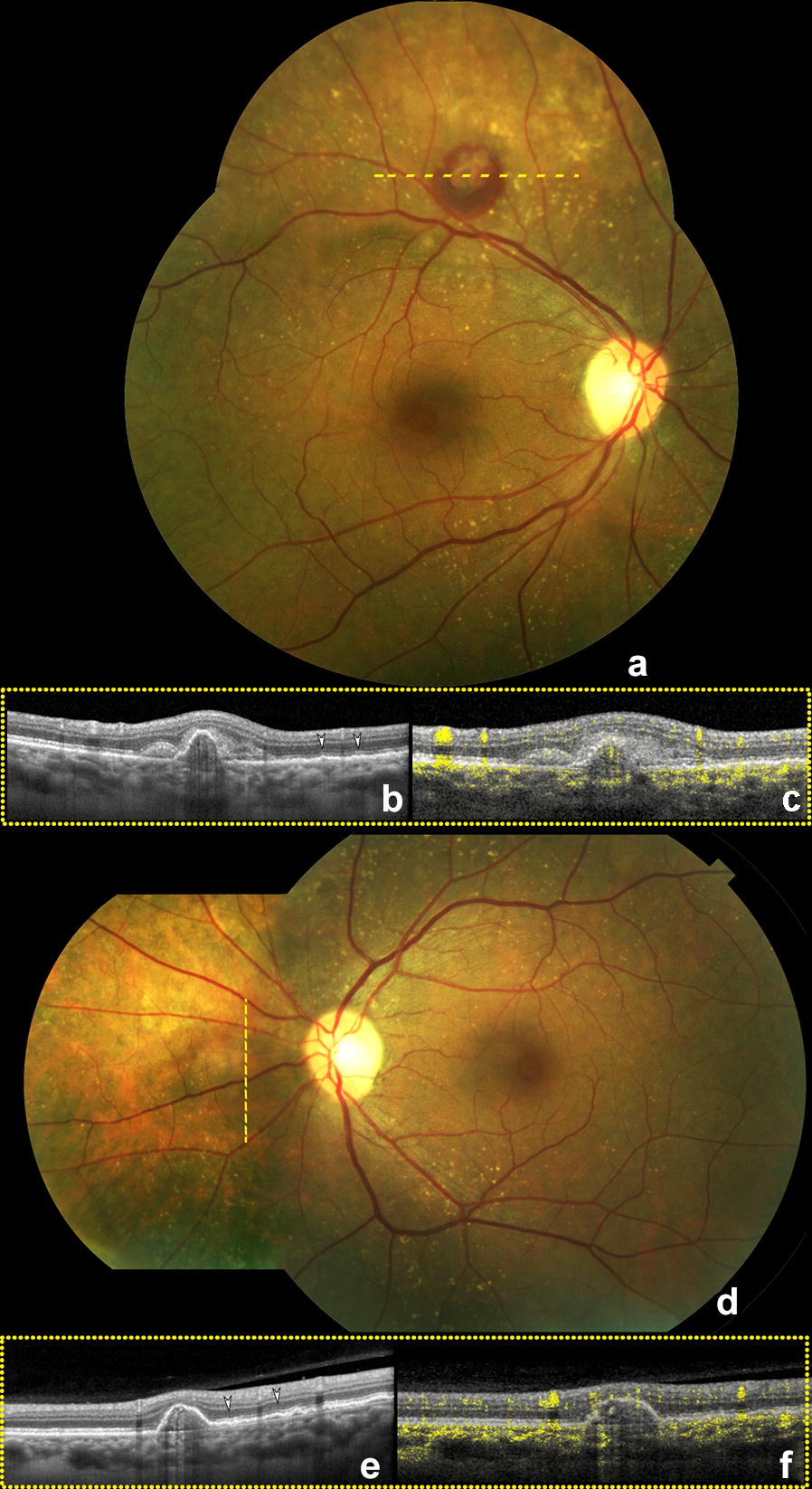
Color montage photographs and optical coherence tomography angiography. Color montage photograph of the right eye **a** shows an orange pigment epithelium detachment (PED) with a hemorrhage visible just above the superotemporal vascular arcade. Spectral-domain optical coherence tomography (SD-OCT) B-scan (**b**), obtained along the lesion as indicated by the yellow dashed line reveals retinal pigment epithelium (RPE) irregularities as indicated by white arrowheads. Optical coherence tomography angiography (OCTA) B-scan with angiographic flow overlay **c** shows flow signal within the PED. Color montage photograph of the left eye **d** shows a nasal PED. SD-OCT B-scan **e** along the dashed line demonstrates a peaked PED with a shallow irregular portion (white arrowheads). OCT B-scan with flow overlay **f** of the same area reveals flow signal beneath the RPE

**Fig. 2:**
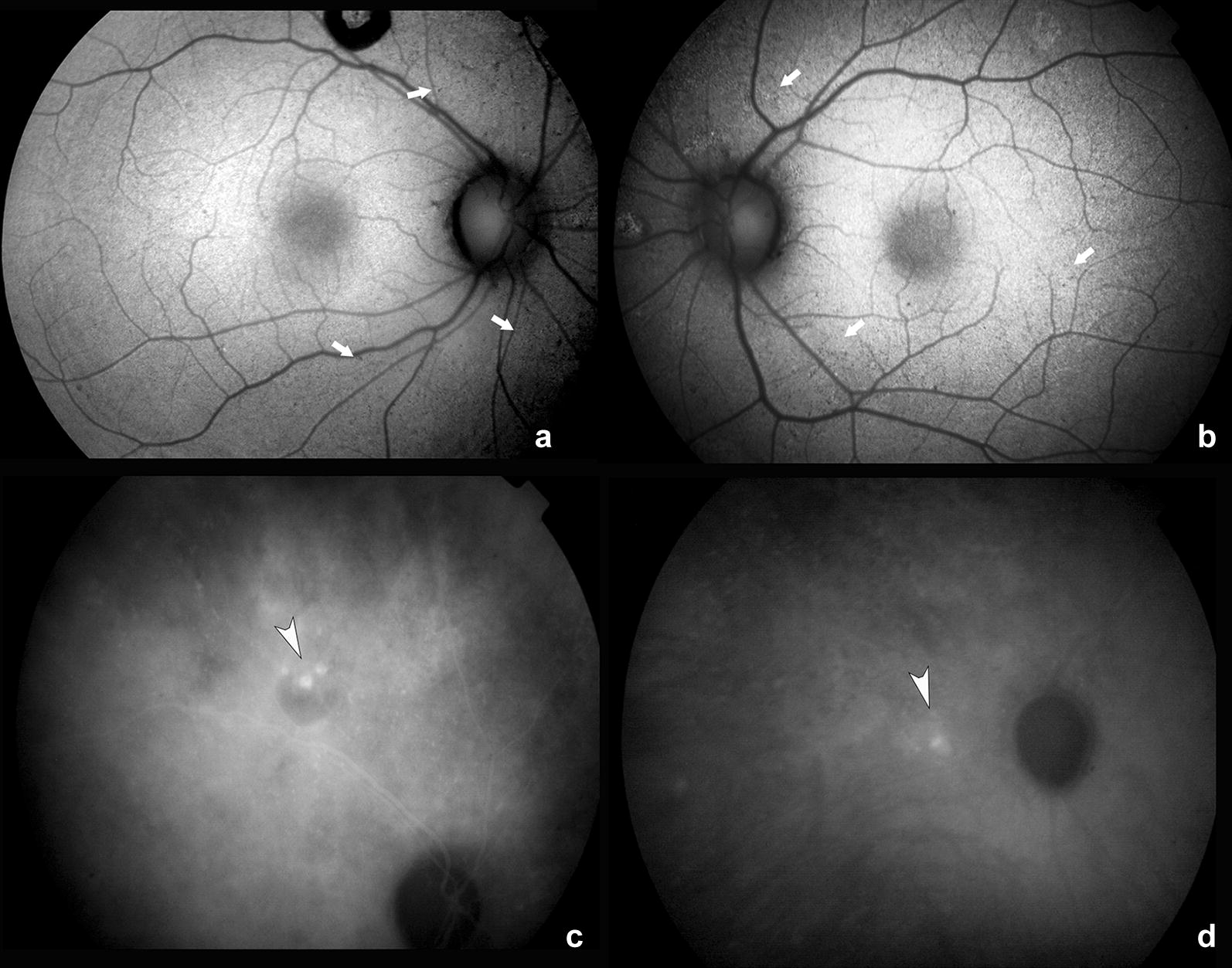
Fundus autofluorescence and indocyanine green angiography. Fundus autofluorescence shows the presence of cuticular drusen scattered along the vascular arcades and in proximity to the lesion (white arrows*)* in both right (**a**) and left (**b**) eyes. Indocyanine green angiogram confirmed the presence of multiple hyperfluorescent nodular lesions (white arrowheads) with hypofluorescent halos in the right eye (**c**) and faint surrounding hyperfluorescence in the left eye (**d**)

A 61-year-old white woman with an unremarkable medical history was referred for an evaluation of an asymptomatic retinal hemorrhage detected in her right eye. Her past ocular history was significant for a complete posterior vitreous detachment in the right eye. Her best-corrected visual acuity was 20/20 in each eye. Anterior segment examination and applanation tensions were unremarkable. Ophthalmoscopic examination of the right eye identified subretinal hemorrhage surrounding a pigment epithelial detachment (PED) located above the superotemporal vascular arcade (Fig. 1a). Spectral-domain optical coherence tomography (SD-OCT) demonstrated the presence of a PED accompanied by irregularities of the retinal pigment epithelium (RPE) profile (Fig. 1b). Optical coherence tomography (OCT) B-scan with angiographic flow overlay showed a peaked PED with intrinsic flow signal (Fig. 1c). Ophthalmoscopic examination of the left eye demonstrated a PED nasal to the optic nerve (Fig. 1d) characterized by a dome-shaped elevation of the RPE with a shallow irregular portion on SD-OCT (Fig. 1e), whereas the angiographic flow overlay revealed intrinsic flow signal (Fig. 1f). Multiple, small, cuticular drusen appearing as small hypoautofluorescent dots on fundus autofluorescence (FAF) were seen in both eyes along the vascular arcades (Fig. 2a, b). Indocyanine green angiography (ICGA) showed focal areas of hyperfluorescence within the PEDs, indicative of AT1 (Fig. 2c, d).

**Fig. 3:**
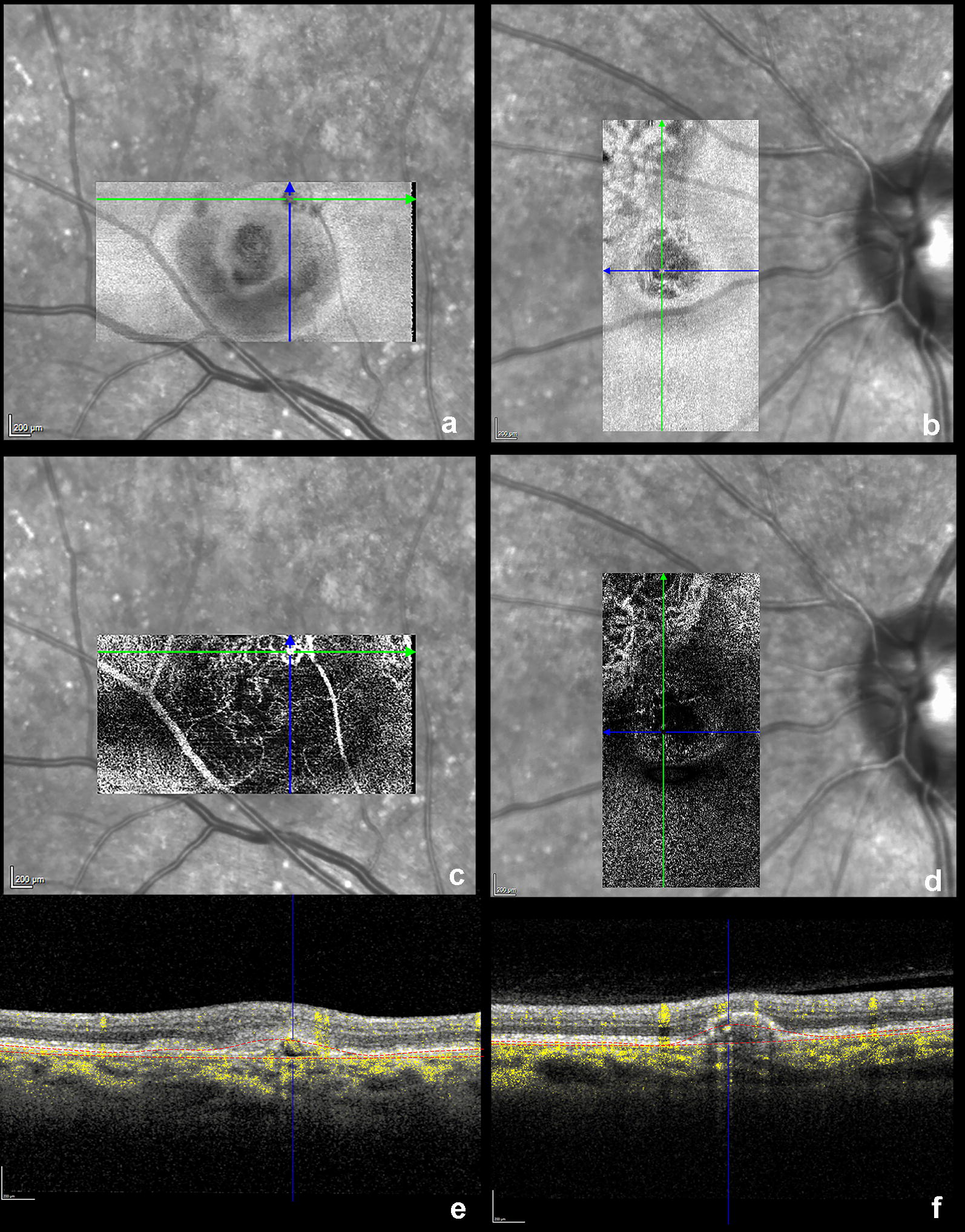
Optical coherence tomography angiography (OCTA) with the right eye represented by the right column (**a**, **c**, **e**) and the left eye by the left column (**b**, **d**, **f**). The structural slabs **a**, **b** of the lesions in both eyes were obtained using an RPE-RPE fit segmentation. *En face* OCTA with customized segmentation between RPE and Bruch’s membrane shows a type 1 neovascular network with evident aneurysmal dilatation, as demarcated by the crossing blue and green lines (**c**, **d**). Dense B-scan with angiographic flow overlay **e**, **f** demonstrates the corresponding cross-sectional view and the customized segmentation of the respective lesions

OCT angiography *en face* slab demonstrated aneurysmal dilatation arising from a type 1 neovascular network, particularly evident in the right eye (Fig. 3a, c, e) with active lesions. As comparative imaging from the patient’s prior examination showed increased hemorrhage, treatment with anti-vascular endothelial growth factor (VEGF) therapy was initiated for the right eye at that time.

## Discussion and conclusions

Cuticular drusen are sub-RPE deposits, visible as small, pale yellow spots distributed in various patterns throughout the fundus. Fluorescein angiography and fundus autofluorescence are both useful for confirming their presence [6]. Recently, Balaratnasingam et al. [2] reported findings in 240 eyes with cuticular drusen from 120 patients (mean age = 57.9 years) having a mean follow-up of 3.7 years. Macular neovascularization was detected in 30 eyes (12.5%), with no cases demonstrating findings consistent with AT1 [2]. In our literature review, we could not identify prior reports of AT1 occurring in association with cuticular drusen, but since 76.7% of the eyes with macular neovascularization in Balaratnasingam et al. [2] cohort had type 1 lesions, the occurrence of AT1 is not unexpected.

Pachychoroid neovasculopathy was first described by Pang and Freund as type 1 neovascularization occurring over areas of increased choroidal thickness and dilated choroidal vessels [7]. Pachychoroid neovasculopathy is often associated with saccular dilatations arising from sub-RPE neovascular tissue, similar to aneurysmal lesions, best seen with OCT and ICGA [3, 7–9]. The prevalence of these PCV/AT1 lesions in Asian populations have ranged from 24.6 to 54.7% [10–12]. In our case, the peaked PED was associated with a shallow irregular RPE elevation in both eyes, and the *en face* OCTA confirmed the presence of aneurysmal dilatations arising from type 1 neovascularization. In fact, it has been reported that flat or shallow irregular PEDs reveal type 1 vascular networks in 95% of cases in eyes with pachychoroid neovasculopathy [13, 14].

However, in conflict with the classic definition of PCV [5], the presence of ‘polypoidal’ dilations have also been recognized in association with central serous chorioretinopathy, myopic staphyloma, radiation retinopathy, and angioid streaks [9, 15–19]. More recently, the first case of AT1 associated with type 1 neovascularization secondary to AMD and sero-hemorrhagic PED was described histopathologically. This case presented important analogies with our case, but more importantly it confirmed the presence of the neovascularization and associated aneurysmal dilations above Bruch’s membrane in the context of typical AMD and type 1 neovascularization [20]. Taken together, these observations further support the use of the more comprehensive term “aneurysmal type 1” when describing an aneurysmal variant of type 1 neovascularization, regardless of the associated pathology. Thus this report of AT1 in yet another retinal disease producing type 1 neovascularization further strengthens the characterization of AT1 as variant of sub-RPE neovascular proliferation rather than as a specific disease limited to certain races. Further studies would be desirable to understand therapeutic and prognostic implications in this particular form of neovascularization accompanied by aneurysmal dilatations.

## Abbreviations

AT1: aneurysmal type 1 neovascularization
PCV: polypoidal choroidal vasculopathy
PED: pigment epithelial detachment
FAF: fundus autofluorescence
ICGA: indocyanine green angiography
